# Terrestrial carbohydrates support freshwater zooplankton during phytoplankton deficiency

**DOI:** 10.1038/srep30897

**Published:** 2016-08-11

**Authors:** Sami J. Taipale, Aaron W. E. Galloway, Sanni L. Aalto, Kimmo K. Kahilainen, Ursula Strandberg, Paula Kankaala

**Affiliations:** 1 Lammi Biological Station, University of Helsinki, Pääjärventie 320, FIN-16900 Lammi, Finland; 2 Department of Environmental and Biological Sciences, University of Eastern Finland, P.O. Box 111, 80101 Joensuu, Finland; 3 Oregon Institute of Marine Biology, University of Oregon, P.O. Box 5389, Charleston, Oregon, USA; 4 Department of Biological and Environmental Science, University of Jyväskylä, P.O. Box 35 (YA), 40014 Jyväskylä, Finland; 5 Kilpisjärvi Biological Station, University of Helsinki, Käsivarrentie 14622, FIN-99490 Kilpisjärvi, Finland; 6 Department of Environmental Sciences, University of Helsinki, P.O. Box 65, 00014 University of Helsinki, Finland

## Abstract

Freshwater food webs can be partly supported by terrestrial primary production, often deriving from plant litter of surrounding catchment vegetation. Although consisting mainly of poorly bioavailable lignin, with low protein and lipid content, the carbohydrates from fallen tree leaves and shoreline vegetation may be utilized by aquatic consumers. Here we show that during phytoplankton deficiency, zooplankton (*Daphnia magna*) can benefit from terrestrial particulate organic matter by using terrestrial-origin carbohydrates for energy and sparing essential fatty acids and amino acids for somatic growth and reproduction. Assimilated terrestrial-origin fatty acids from shoreline reed particles exceeded available diet, indicating that *Daphnia* may convert a part of their dietary carbohydrates to saturated fatty acids. This conversion was not observed with birch leaf diets, which had lower carbohydrate content. Subsequent analysis of 21 boreal and subarctic lakes showed that diet of herbivorous zooplankton is mainly based on high-quality phytoplankton rich in essential polyunsaturated fatty acids. The proportion of low-quality diets (bacteria and terrestrial particulate organic matter) was <28% of the assimilated carbon. Taken collectively, the incorporation of terrestrial carbon into zooplankton was not directly related to the concentration of terrestrial organic matter in experiments or lakes, but rather to the low availability of phytoplankton.

Primary production is a key process in the biosphere that synthesizes organic compounds (monosaccharides, amino acids, and fatty acids) fueling biomass production and function of organisms across the food webs. Both marine and freshwater food webs are rich in essential ω-3 and ω-6 polyunsaturated fatty acids (PUFA; refs 1,2), whereas terrestrial food webs are low in physiologically important eicosapentaenoic acid (EPA, 20:5ω3) and docosahexaenoic acid (DHA, 22:6ω3)^3^. Lipids and essential fatty acids (FA) synthesized by aquatic primary producers (phytoplankton, littoral algae) not only support aquatic food webs^4^,^5^,^6^, but also many insect, bird and mammalian species living at the interface of aquatic and terrestrial ecosystems, highlighting the importance of aquatic support to terrestrial food webs^7^,^8^,^9^. Aquatic subsidies to terrestrial ecosystems can be important since terrestrial food webs (e.g. arthropods) can become lipid-limited^10^. During recent decades, increased loading of terrestrial organic matter to freshwater systems has been detected in several boreal and temperate regions^11^. This may have direct and indirect effects on the availability and quality of diet sources for consumers, such as herbivorous zooplankton and fish in freshwater systems^12^,^13^,^14^. More studies on biochemical composition and assimilation of both terrestrial and aquatic diet sources are needed for understanding costs and benefits for aquatic consumers utilizing terrestrial resources.

Somatic growth and reproduction of consumers is strongly affected by the quality of lipids and proteins, specifically by the concentration and composition of ω-3 and ω-6 PUFA and amino acids^2^. Additionally, non-essential biomolecules such as carbohydrates are important dietary energy sources for consumers, which can save more valuable biomolecules, such as proteins for reproduction and somatic growth. This phenomenon is called “protein sparing” and is found in a wide range of organisms^15^,^16^. However, carbohydrates can only fulfill a consumer’s short-term energy demands, whereas lipids are important as long-term energy storage and consumers with high lipid content, in general, have high energy density. Lipid energy storage is important for some zooplankton taxa (e.g., *Eudiaptomus sp., Limnocalanus macrurus*, ref. 17) for over winter survival. In addition, the deficit of dietary lipids has shown to decrease somatic growth and reproduction in consumers at different trophic levels in the food chain up to predators (e.g. beetles, seabirds, sea lions; refs 18, 19, 20).

The role of terrestrial organic matter in supporting aquatic food webs (allochthony) is estimated to be significant in brown-water lakes and headwater rivers^21^,^22^,^23^, which receive large inputs of terrestrial organic matter from the catchment^24^,^25^. In food web studies based on stable carbon and/or hydrogen isotopes, dietary sources of consumers are often treated as biochemically homogenous groups (autochthonous or allochthonous), which may be difficult to separate due to overlapping isotope values, and rely on many assumptions in calculations for phytoplankton fractionation and ‘environmental water’^26^,^27^,^28^. Moreover, the isotope values of consumers are combinations of different organic carbon molecules (proteins, saccharides and lipids) often originating from distinct dietary sources^29^.

More than >90% of the allochthonous, terrestrial organic matter in aquatic ecosystems is in dissolved form and consists mainly of poorly bioavailable recalcitrant humic substances^30^,^31^. Only a small fraction (<20%) of the dissolved organic carbon (DOC) in freshwater systems consists of biodegradable low-molecular weight fraction, such as organic acids, free amino acids and carbohydrates^32^,^33^. These biodegradable molecules may maintain heterotrophic bacterial production and potentially support the higher trophic levels^34^. Many cladoceran zooplankton taxa are capable of utilizing bacteria as a dietary resource^35^,^36^. However, due to lack of essential PUFA and sterols, bacteria are not a nutritionally adequate resource, and cannot solely support somatic growth and reproduction of cladoceran zooplankton^37^,^38^.

In addition to bacteria, detrital particulate organic matter from terrestrial ecosystems, e.g., originating from fallen tree leaves or shoreline vegetation, can be directly utilized by primary consumers^39^. Terrestrial particulate organic matter (t-POM) in lakes is highly degraded and is mostly composed of lignin and cellulose, containing only trace amounts of fatty acids and sterols^12^,^40^,^41^. The predominant class of fatty acids in t-POM is the long chain saturated fatty acids (LC-SAFA) (>60%), whereas the proportion of PUFA (predominately α-linolenic acid, ALA, 18:3ω3) is <1% 41. Herbivorous zooplankton (crustacean *Daphnia*) can utilize t-POM, but as a sole diet source it yields poor growth and reproduction^12^,^38^,^42^.

In the current study, we combined experimental and field data to assess the importance of t-POM to cladoceran zooplankton. Based on previous experimental and field studies (e.g., Taipale *et al.* 2008, Galloway *et al.* 2014) we tested two hypotheses: 1) terrestrial-origin carbohydrates and proteins are assimilated efficiently and support biomass growth of cladoceran zooplankton, and 2) the utilization t-POM and bacteria by zooplankton is related to the concentration of allochthonous organic carbon in the lakes. Firstly, we analyzed biochemical composition of major potential aquatic (phytoplankton) and terrestrial food sources (t-POM of wetland reed and deciduous tree leaves) available for herbivorous zooplankton. Secondly, we tested how different levels of t-POM affect somatic growth of a model consumer organism, the cladoceran crustacean *Daphnia magna*, using these t-POM types and phytoplankton as diet sources as well as to estimate threshold values for autochthonous essential biomolecules support needed to maintain the optimal growth. We analyzed how the proportions of different biomolecules (carbohydrates, lipids, proteins) in the total body carbon of *Daphnia* changed with increasing diet allochthony and investigated if *Daphnia* was able to bioconvert terrestrial-originated carbohydrates to fatty acids. Thirdly, we studied zooplankton dietary assimilation in 16 boreal and in 5 subarctic lakes using fatty acid based mixing model calculations^38^,^43^, to identify the importance t-POM subsidy in different lake types.

## Results

### Quality of phytoplankton and t-POM

**Figure 1:**
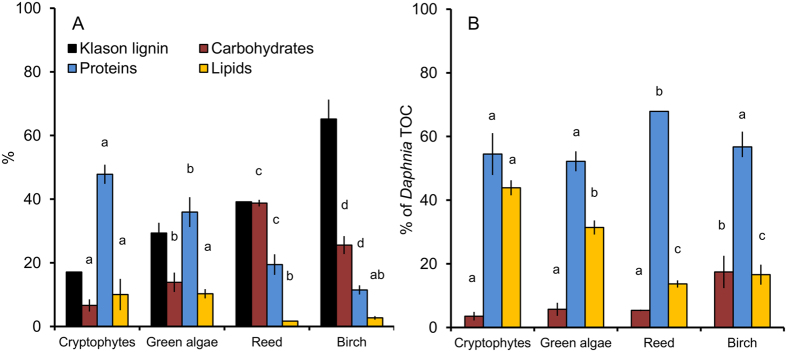
(**A**) Biochemical content (mean ± SD% of total organic carbon, TOC) of cryptophytes (*Cryptomonas ozolinii*), and green algae (*Acutodesmus* sp.), particles of dried birch (average of *Betula pedula* and *B. pubescens czerepanovii*) and reed (*Phragmites australis*) leaf litter and (**B**) corresponding values of *Daphnia* fed with those diets. Different letters (a–d) denote significant differences in carbohydrate, protein and lipid content between diet sources (**A**) and *Daphnia* fed solely with those diets (**B**).

The biochemical composition of phytoplankton and t-POM, originating from tree and reed leaf litter, showed clear qualitative differences (Fig. 1A, Table 1). A high proportion of the leaves consisted of lignin (birch 65 ± 7%, reed 39 ± 0.1% of total organic carbon (TOC). Total carbohydrate content of reed leaves (>75% consisting of glucose) was significantly higher (39 ± 1% of TOC) than that of tree leaves (29 ± 5% of TOC) and phytoplankton (7–10% of TOC). In phytoplankton, the proportions of proteins and lipids of TOC were greater than in tree and reed leaves (Fig. 1A, Table 1).

### The fate of terrestrial carbohydrates, lipids and proteins in zooplankton

Our laboratory experiments showed that herbivorous zooplankton (*Daphnia*) fed on phytoplankton had significantly higher lipid content (Cryptophyte diet 44 ± 2%, green algal diet 31 ± 3% of TOC) than those fed solely on terrestrial diets (14–17% of TOC) (Fig. 1B). The contribution of carbohydrates to *Daphnia* (Fig. 1B) was higher in those fed on birch leaf particles (15 ± 3% of TOC) compared with those with the other diets. About half of the organic carbon in *Daphnia* consisted of proteins, the proportion being highest with reed diet (Fig. 1B).

**Figure 2:**
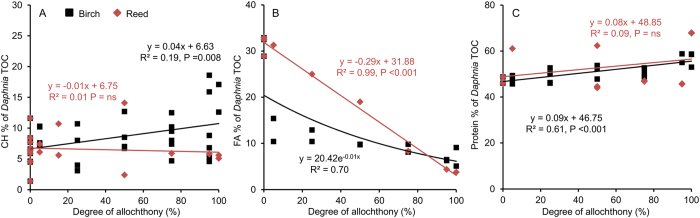
(**A**) Proportion of carbohydrates, (**B**) fatty acids (FA) and (**C**) proteins in *Daphnia* body carbon (% of TOC) grown with diets mixed with *Acutodesmus* green alga and birch or reed particles. Best fit linear or exponential model equations with R^2^ and P values are also shown.

Along with increasing allochthony with the mixed diet of *Acutodesmus* green alga and t-POM, no trend in carbohydrate content was seen in *Daphnia* fed with reed leaf particle mixtures (Fig. 2A). In *Daphnia* fed with birch leaf particle mixtures, the proportion of carbohydrates of TOC increased significantly with allochthony, although the variation was rather high (Fig. 2A). The proportion of fatty acids in *Daphnia* decreased significantly with the diet allochthony both with birch and reed particle mixed diets (Fig. 2B). Until ca. 90% allochthony, the fatty acid content was higher in those fed with reed mixed particles compared with the birch treatment. The contribution of proteins increased slightly with allochthony gradient with birch mixed particles, while in reed treatment the variation between replicates was too high to produce a significant regression (Fig. 2C).

**Figure 3:**
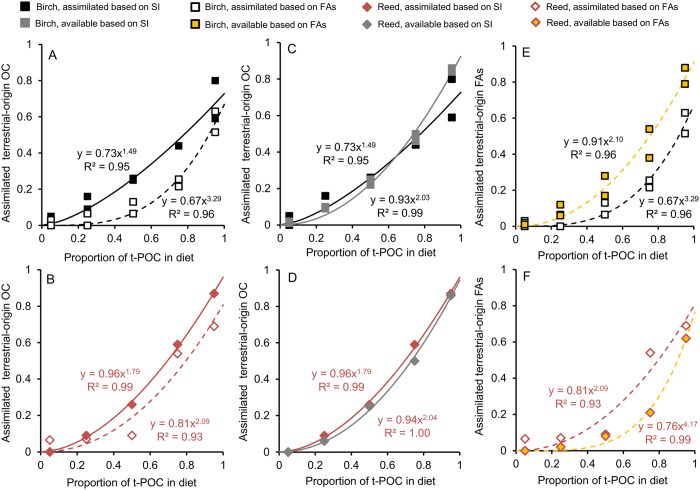
Assimilation of terrestrial-origin carbon by *Daphnia* in relation to the proportion of terrestrial particulate organic carbon (t-POC) in (**A**) mixed birch-algae diet (square) and (**B**) in mixed reed-algae diet (diamond). The assimilation of terrestrial-origin organic carbon (OC) (filled black and red symbols), based on stable isotope analyses, did not differ to that available in the diets (filled grey symbols; (**C**,**D**). The proportion of assimilated terrestrial-origin fatty acids (FAs) by *Daphnia* (white squares) was lower than that available in mixed birch-algae diet (yellow squares) but higher (white diamonds) than that available in mixed reed-algae diet (yellow diamonds; **E**,**F**).

The proportion of assimilated terrestrial organic carbon increased with the proportion of t-POM in the diets. The contribution of assimilated terrestrial organic carbon was greater based on stable isotopes than on fatty acids in both terrestrial diets (Fig. 3A,B), but the difference was greater in the birch experiment. Based on stable isotopes, the assimilated proportion of terrestrial-origin organic carbon was similar than that available in the diet with both t-POM sources (Fig. 3C,D). In the reed experiment, the proportion of assimilated saturated fatty acids in *Daphnia* exceeded that of available in the diet (Fig. 3F), whereas in the birch experiment, the proportion of assimilated saturated fatty acids was lower than what was available in t-POM (Fig. 3E). Thus, *Daphnia* fed on reed leaf particles had 6-times higher fatty acid assimilation rate relative to the ones fed on birch leaf particles.

**Figure 4:**
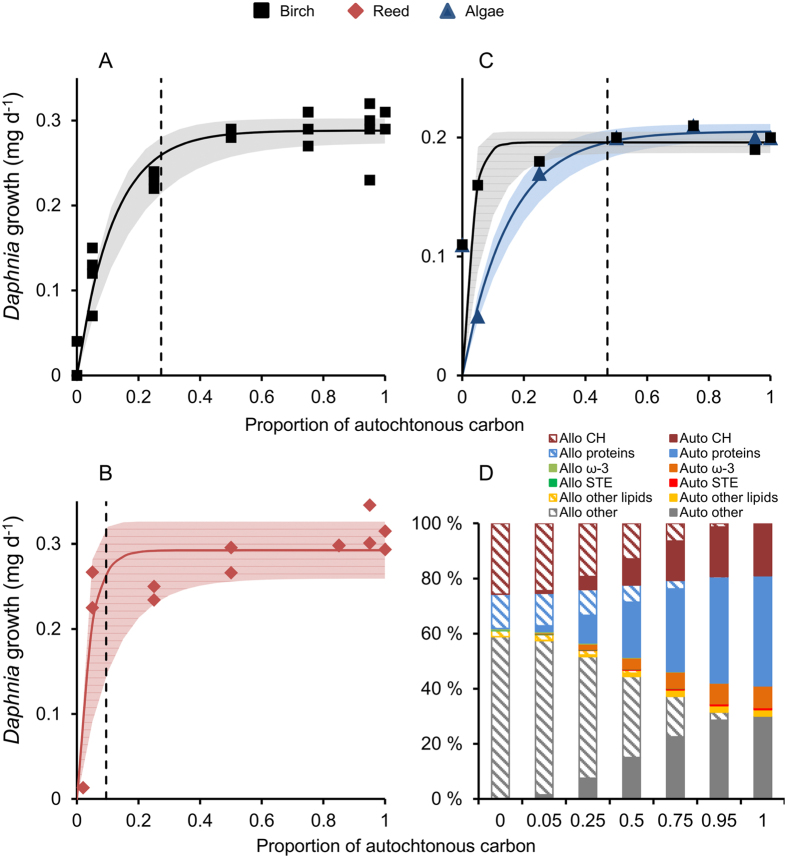
Somatic growth rate of *Daphnia* in relation to the proportion of autochthonous carbon during a 10-day experiment, fed with (**A**) mixed birch-algae diet (square), and (**B**) with mixed reed-algae diet (diamond) in non-limiting food conditions (5 mg C L^−1^). Dashed lines refer to optimal growth rate threshold (or 90% of maximal somatic growth). (**C**) Biomass growth rate of *Daphnia* in relation to the proportion of autochthonous carbon during a 21-day experiment fed with pure green algae (*Acutodesmus* sp.; triangle) in food gradient from 0.25 to 5 mg C L^−1^ and with mixed diet of green algae and silver birch leaf particles (total food concentration 5 mg C L^−1^; square). *Daphnia* benefitted from additional terrestrial carbon when algal food concentration was < 2.4 mg C L^−1^. Dashed line refers to threshold point where terrestrial addition did not benefit *Daphnia* anymore. Shaded areas in A, B and C are the 95% confidence intervals. (**D**) The proportions of biochemical compounds in *Daphnia* diet along with decreasing allochthonous (allo) and increasing autochthonous (auto) carbon. Pure terrestrial diet mainly consisted of carbohydrates (CH) and undigestible compounds (others), whereas pure autochthonous diet had high protein and lipid content. (Symbols: ω-3 = ω-3 fatty acids, STE = sterols).

In the 10-day experiment, *Daphnia* achieved optimal somatic growth rate (90% of maximal somatic growth) when the proportion of autochthonous (*Acutodesmus*) carbon was ≥27% and ≥9.5% of total dietary carbon in birch-algae and reed-algae diet mixtures, respectively (dashed lines in Fig. 4A,B). In terms of biochemical content, this meant that *Daphnia* required 108 μg of ω-3 fatty acids mg C^−1^, 11 μg of sterols mg C^−1^ and 0.5 mg proteins mg C^−1^ from *Acutodesmus* to achieve optimal somatic growth in the birch-algae experiment. The autochthonous supplements needed in reed-algae experiment were much smaller: 37 μg of ω-3 fatty acids mg C^−1^, 4 μg of sterols mg C^−1^, and 0.2 mg proteins mg C^−1^. In the second experiment lasting 21 days, *Daphnia* were cultured either solely with *Acutodesmus* concentration gradient from 0.25 to 5 mg C L^−1^ or with the same concentration gradient of *Acutodesmus*, but supplemented with birch leaf particles up to concentration 5 mg C L^−1^ (Fig. 4C). This experiment demonstrated that when the proportion of autochthonous algae in *Daphnia* diet was less than 47% of total carbon content (equals to < 2.4 mg C L^−1^), somatic growth of *Daphnia* benefitted from birch leaf particles (Fig. 4C). At this threshold 7% of ω-3 fatty acids, 23% of sterols, 60% of carbohydrates and 26% of proteins in the diet were of terrestrial origin (Fig. 4D).

### Cladoceran allochthony in lakes

**Figure 5:**
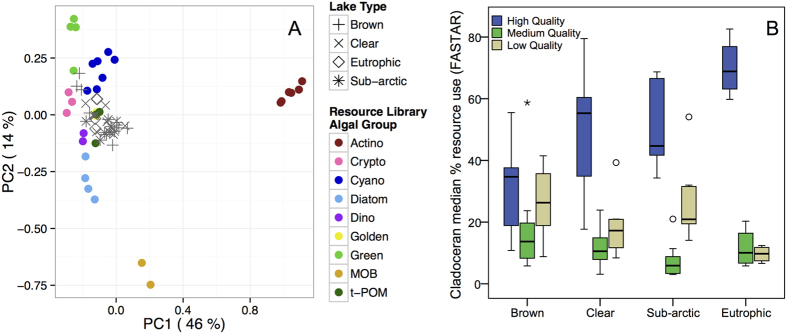
(**A**) Principal components analysis (PCA) visualizing the multivariate fatty acid resource-library of experimentally fed *Daphnia* fed known basal end-members and the wild cladocerans collected in the study lakes. Each colored filled circles is the mean fatty acid profile of *Daphnia* fed a different basal taxon within each of the nine end-member groups [abbreviations: Actino (Actinobacteria); Crypto (Cryptophyceae); Cyano (Cyanophyceae); Diatom (Heterokontophyta); Dino (Dinophyceae); Golden (Synurophyceae); Green (Cryptophyceae); MOB (methane oxidizing bacteria); t-POM (terrestrial particulate organic matter, Plantae)]. Gray symbols are the fatty acid profiles of wild cladocerans collected in each of the 4 lake types. The total variation explained by each PC is displayed on the axes. PC3 (not presented) explained 12% of the total variation. (**B**) The contribution (Boxplots with median, 25^th^ and 75^th^ percentiles, with whiskers for 5^th^ and 95^th^ percentiles) of diet nutritional quality for *Daphnia* was divided into high (phytoplankton with EPA), intermediate (phytoplankton with high levels of ALA) and poor (low content of ω-3 fatty acids; Actinobacteria, MOB and t-POM) quality diets, as modeled by FASTAR.

The sampled wild zooplankton from all lakes fell inside of the multivariate resource-polygons of the resource library of the fatty acid profiles of *Daphnia* fed end members in controlled feeding trial (Fig. 5A), a prerequisite best practice for bio-tracer based mixing model analysis^43^,^44^. The contribution of high-quality phytoplankton (rich in EPA; ref. 12) diets in cladocerans differed among lake types (ANOVA, F_3,27_ = 6.97, p = 0.001), and appeared to be a less important dietary resource for cladocerans in brown-water lakes as compared with the other lake types (Fig. 5B). In clear-water, subarctic and eutrophic lakes 50–70% of cladoceran diets consisted of high-quality phytoplankton, the greatest proportion appearing in the eutrophic lakes. In the utilization of high-quality resources, post-hoc tests found significant differences between brown-water lakes and eutrophic lakes (p = 0.001), but differences were not statistically significant between brown-water and clear or subarctic lakes. The proportion of medium-quality (phytoplankton rich in ALA; 18:3ω3) diets did not differ among lake types (ANOVA, F_3,27_ = 1.48, p = 0.243), but support from low-quality diets (the lowest content of ω-3 fatty acids, i.e. t-POM and bacteria) did differ among lake types (ANOVA, F_3,27_ = 3.72, p = 0.023). However, post-hoc tests between lake types for low-quality diets did not differ (Fig. 5B).

**Figure 6:**
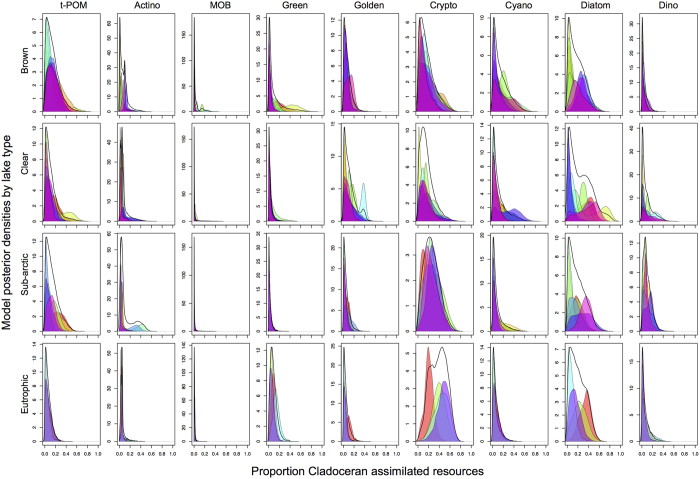
FASTAR mixing model estimates of the proportional assimilation of basal resources by wild cladocera from 9 basal resource groups at the four lake types (rows). The model was run using all 24 fatty acids in the profiles of *Daphnia* fed known end-member diets (see Methods; Fig. 4A) in independent feeding trials. Each plot shows the posterior density (y-axis) of results from the mixing model for a given dietary source (x-axis). The FASTAR solution for *Daphnia* from each individual lake are reported as different colored distributions for each replicate [brown lakes (n = 10); clear lakes (n = 10); subarctic lakes (n = 7); and eutrophic lakes (n = 4)], along with median ‘lake-level’ solution compiled from all replicates within that lake type (non-colored distribution). Resource group names (columns) follow abbreviations defined in the Fig. 5 caption.

Terrestrial particulate organic matter (t-POM) contributed approximately 11 ± 6% (Mean ± SD of median values), but varied between lakes (Fig. 6, Table 2). Assimilated t-POM content differed among lake types (ANOVA, F_3,27_ = 5.45, p = 0.005), being higher (15 ± 7% of all) in brown-water, clear-water (10 ± 5% of all), and subarctic lakes (14 ± 3% of all) than in the eutrophic lakes (3 ± 2% of all). Correspondingly, the sum of autochthonous phytoplankton consisted in average 64 ± 24% of cladoceran diets and the proportion differed among the lake types (ANOVA, F_3,27_ = 4.96, p = 0.007), with eutrophic lakes having the highest (86 ± 5%) phytoplankton assimilation by zooplankton (Fig. 6). Cryptophytes and diatoms were the most important dietary resources in all lake types. Cryptophytes were the major dietary resource for cladocerans in eutrophic (49 ± 16% of all) and subarctic lakes (28 ± 11% of all), whereas diatoms were the major dietary source in clear-water (22 ± 11% of all) and brown-water lakes (15 ± 11% of all). Additionally, green algae contributed 12 ± 14% of all diet sources in brown-water lakes and dinoflagellates contributed 13 ± 15% of all diets in clear-water lakes.

The proportion of methane oxidizing bacteria (MOB) in cladoceran diets was generally low (<2%) with the exception of two small brown-water lakes (Mekkojärvi and Horkkajärvi, Table 2). The proportion of Actinobacteria in the cladoceran diet varied within and between lakes, maximum in the subarctic Lake Kuohkimajärvi (32 ± 12%). If all Actinobacteria are assumed to be 100% supported by allochthonous organic carbon, the average zooplankton allochthony would have been 19 ± 10%. The proportions of t-POM and Actinobacteria in the zooplankton diets were not correlated with water colour, DOC, nitrogen, phosphorus or chlorophyll-*a* concentration of the lakes (r < 0.1, P > 0.05).

## Discussion

Our field and laboratory study of herbivorous zooplankton demonstrates that although freshwater ecosystems are strongly affected by their catchment, terrestrial organic matter can only limitedly support zooplankton production and its contribution varies greatly among lakes. We show that the assimilation of terrestrial organic carbon is not directly related to its availability, but rather to the lack of better quality diets. We demonstrated that this is due to the fact that terrestrial plant litter entering lakes contains high amounts of biologically unavailable lignin, and more carbohydrates, but less proteins and lipids (including essential fatty acids and amino acids) per carbon unit than aquatic primary consumers require for their optimal growth and reproduction. In the absence of phytoplankton and under circumstances of forced high allochthony, the carbohydrate content of herbivorous zooplankton increased from ~6 even up to 10% of TOC, reflecting the poor biochemical quality of terrestrial organic matter. We found that when algal food availability is low, t-POM supplements benefit somatic growth of *Daphnia*, thus, partly supporting our first hypothesis. However, due to the low-lipid and low-protein content, terrestrial organic carbon could support growth only up to a certain threshold, indicating that herbivorous zooplankton have to satisfy the most essential biochemical demands mainly with phytoplankton (e.g., refs 42 and 45).

Leaf litter of common deciduous trees in the North-America and Scandinavia consists mainly of lignin, which is non-digestible for zooplankton, and is a poor source for proteins, ω-3 FA and sterols, shown by our biochemical analyses (this study, refs 12 and 38). Carbohydrates are the most beneficial compounds of t-POM, which can be utilized by aquatic primary consumers. Due to their higher carbohydrate and protein content, the quality of reed leaf particles was a better dietary resource for herbivorous cladocerans than that of tree leaves. In fact, *Daphnia* fed with mixed reed-algae diet had a higher somatic growth rates than those grown with mixed birch-algae diet. Glucose is the major oligosaccharide in the leaves, suggesting that t-POM can be an optimal short-term energy source for zooplankton. This was observed in our 20-day laboratory experiments, where birch supplementation enhanced *Daphnia* growth significantly when phytoplankton concentration was low. The results show that *Daphnia* uses a ‘sparing strategy’ in circumstances of high carbohydrate, but low lipid and protein availability to maximize its somatic growth. Thus, *Daphnia* is able to use terrestrial carbohydrates for energy and save proteins (amino acids) and lipids (fatty acids) for structural components.

*Daphnia* fed on tree leaf particles had ~twice higher carbohydrate content than what was found in those fed on reed. This, together with low ω-3:ω-6 –ratio of tree leaves^12^,^38^,^41^, indicated higher nutritional stress in *Daphnia* using the birch diet than in those using reed or phytoplankton diets. Furthermore, the assimilated proportion of terrestrial-origin fatty acids by *Daphnia* in the reed experiment was higher than the proportion available in the diet, suggesting that *Daphnia* is able to convert excess carbohydrates from reed leaves to fatty acids. However, this was not observed in *Daphnia* with birch leaf diet, for which the lower proportion of carbohydrates in the diet possibly forced *Daphnia* to use both terrestrial-origin carbohydrates and fatty acids to meet its energy demand. Since the carbon isotope signal comes from all organic compounds, the difference between stable isotopes and fatty acids (two source mixing model) results is due to the utilization of terrestrial-origin proteins and carbohydrates. The difference between the methods was smaller in the reed diet than in the birch diet even though the reed diet initially contained less fatty acids than birch diets. Therefore, *Daphnia* has likely obtained fatty acids by conversion from carbohydrates in the reed experiment. Our estimates of t-POM utilization by herbivorous zooplankton in 21 lakes based on fatty acid mixing model (FASTAR) results are within the range of zooplankton allochthony estimates for temperate and boreal lakes based on stable carbon (δ^13^C) and/or hydrogen (δD) isotope analyses^46^,^47^,^48^. In stable isotope mixing-model calculations phytoplankton is generally assumed to be one solid group, because although carbon isotope values can differ greatly between taxa^49^,^50^, this is rarely if ever measured. This problem does not occur in fatty-acid based modeling since group-specific fatty acid characteristics of phytoplankton and other resources are included in the resource library of the model. The fatty acid mixing model analyses (FASTAR) show that in all four lake types, the high-quality phytoplankton (cryptophytes and diatoms) form the base of herbivorous zooplankton diet. Use of these high quality resources, which are rich in essential amino acids, fatty acids, and sterols^51^,^52^. Also makes somatic growth and reproduction for zooplankton possible during utilization of lower quality diets^12^,^37^.

Our second hypothesis, that the utilization t-POM and bacteria by zooplankton is related to the concentration of allochthonous organic carbon in the lakes was not supported. We found that the contribution of t-POM in zooplankton diet varies greatly within and among the lakes (from 2 to 27%). Even if >90% of allochthonous carbon inputs is in dissolved form^30^,^31^, suggesting that microbial food chain could be the major link between terrestrial food sources and herbivorous zooplankton^22^, our fatty acid-based mixing model results from 21 lakes indicate that in most cases the contribution of t-POM exceeded that of *Actinobacteria.* This is in agreement with our previous results analyzing cladoceran basal resource support in large boreal lakes^43^. Furthermore, the DOC concentration of lakes did not correlate with the contribution of *Actinobacteria* in the cladoceran diets. Thus, our results suggest that the major pathway of terrestrial organic carbon to zooplankton is not diverted via heterotrophic DOC-utilizing bacteria^53^. Strong positive relationships found between biomasses and production of phytoplankton and bacteria, generally observed in lake ecosystems, suggest that phytoplankton-origin DOC is a very important carbon source for heterotrophic bacteria in all kinds of lakes^54^,^55^. Moreover, the poor growth efficiency of heterotrophic bacteria utilizing terrestrial DOC^56^,^57^ also supports this conclusion. However, the microbial food chain, via bacteria to protozoans, may have some importance in the transfer of terrestrial carbon to higher trophic levels. Here, the quality of organic matter and picoplankton prey available for heterotrophic protozoans affect their quality as diet source to the next trophic level, metazoan plankton^58^. It should also be noted that each additional step in the food chain cause respiratory losses lowering the transfer efficiency of terrestrial carbon to higher trophic levels^44^,^59^.

The high variation in the estimated cladoceran t-POM utilization indicates that when lipid and protein rich autochthonous organic carbon sources are scarce, herbivorous zooplankton can use terrestrial-origin carbohydrates, lipids, and proteins more intensively. This finding is supported by results of 21 lakes, where the assimilation of t-POM was not correlated with DOC concentration, although, in general, higher cladoceran allochthony was detected in the brown-water lakes. For example, in Lake Horkkajärvi, which had the highest DOC concentration of all the sampled lakes, the observed proportion of t-POM assimilated by cladocerans was very low, presumably due to the high densities of better quality food sources (e.g. autotrophic and mixotrophic algae)^48^,^54^ during the sampling season. In small stratified lakes methane-oxidizing bacteria (MOB) can significantly contribute to zooplankton diets^60^,^61^ as found in the two lakes of this study. The trophic pathway from methane to higher trophic levels via MOB may be more related to anaerobic decomposition of fresh, autochthonous organic matter than to allochthonous sources^62^.

Our results are in accordance with recent model results based on field experiments in five lakes^63^, in which zooplankton biomasses and production were low when allochthony exceeded 30%. This was related to the light extinction by brown-coloured terrestrial DOC suppressing phytoplankton primary production and biomass^64^,^65^ available for zooplankton grazers. The high level of zooplankton allochthony seems to be a consequence of the absence of better quality dietary sources rather than the result of the high availability of terrestrial organic matter. Zooplankton allochthony was not the highest in the lakes with the most pronounced loadings of terrestrial matter and this was likely explained by the biochemical composition of t-POM and by nutritional requirements of herbivorous zooplankton. The high estimates of assimilated t-POM by cladoceran zooplankton obtained with the fatty acid mixing model could also indicate low nutritional status of zooplankton in the lakes^41^,^43^.

In conclusion, allochthony of herbivorous zooplankton varies among different type of boreal lakes, and is mainly defined by the availability of lipid and protein-rich phytoplankton. We show that cladocerans use primarily carbohydrates for energy, but can also exploit some lipids and proteins of terrestrial matter for somatic growth. Leaves of shoreline vegetation (reed) had better dietary quality than those of birch, containing less lignin and more glucose, which *Daphnia* was able to partially bioconvert to more usable saturated fatty acids. However, this was not observed in birch diet with low carbohydrate content. Overall, we suggest that relatively high proportion of terrestrial organic carbon in cladocerans and in other aquatic herbivores can result from multiple biochemical processes and that the degree to which organic matter produced by terrestrial plants can support freshwater food webs may depend upon the biochemical content of different terrestrial vegetation. However, the high content of lipids, proteins and other essential biomolecules produced by phytoplankton are needed to sustain all types of aquatic food webs.

## Methods

### Zooplankton and phytoplankton cultures

All experiments were conducted using a clone of *Daphnia magna* (DK-35-9, hereafter *Daphnia*), initially grown and maintained on *Acutodesmus* sp. which was obtained from the Institute of Zoology, University of Basel. We also used *Cryptomonas erosa* (CPCC 466) as high quality diet control in our experiments. *Acutodesmus* sp. was cultured using modified WC solution supplemented with biotin and cyanocobalamin (B_12_)^66^. *Cryptomonas erosa* was cultured using AF6 media^67^. In addition, we cultured some more phytoplankton strains listed in Table 1. Each strain was cultured in a medium specific to that strain (Table 1) and were grown at 20 °C under a 14 h:10 h light:dark cycle with light intensity of 30–70 μmol m^−2^ s^−1^. To obtain differences in carbon isotope signals between the diets, *Acutodesmus* sp. cultures were enriched with ^13^C, 3% of the NaHCO_3_ in the MWC media consisted of NaH^13^CO_3_ (99%), Cambridge Isotope Laboratories].

### Terrestrial carbon source

We used leaf litter particles of common reed [*Phragmites australis* (Cav.) Trin. ex Steud], silver birch (*Betula pendulata*) and arctic birch (*Betula pubescens* subsp. *czerepanovii*) as terrestrial particulate organic matter (hereafter called t-POM) food resources for zooplankton *Daphnia magna*. We collected reed leaves from the shore of Lake Pyhäselkä (eastern Finland), and ground it to small particles using a Fritsch Planetary Mono Mill Pulverisette 6^38^. The particles were then diluted into the WC Media directly and incubated for one month in the. Silver birch and arctic birch leaves were ground to fine particles using a Retch ZM 100 GWB ultra centrifugal Mill^41^. For this experiment ground t-POM was diluted into modified Woods Hole (WC) medium^68^ and filtered through a 50 μm screen and incubated one month in the dark with continuous shaking at 120 rpm.

### Batch experiment

*Daphnia* neonates (~6 h old) were used for all experiments. Neonates from specific adults were divided equally between treatments to minimize maternal effects^69^ and distributed individually into glass vials (40 mL of L16 media) with each treatment consisting of 10 replicates. The media was changed and the *Daphnia* fed every other day with total food concentrations of 1.5, 2 and 5 mg C L^−1^ for ages 2, 4 and 6 + days, respectively. These food concentrations were above the incipient limiting level for ingestion^70^. In a 10-day experiment *Daphnia* was fed with pure (100%) diets of t-POM, and each taxon of phytoplankton (*Cryptomonas erosa* and *Acutodesmus* sp.), and also in gradients of diets consisting 95%, 75%, 50%, 25% and 5% of t-POM and mixed with 5%, 25%, 50%, 75%, 95% of intermediate quality phytoplankton (*Acutodesmus* sp.), respectively, to evaluate how the allochthonous diet impacts on *Daphnia* lipid, protein and carbohydrate content.

### Life table experiment

In the life table experiment lasting 21 days, *Daphnia* were cultured solely with *Acutodesmus* in a food concentration gradient, 0.25, 1.25, 2.5, 3.75, 4.75 and 5 mg C L^−1^, and in parallel cultures with the same concentration gradient of *Acutodesmus*, but added with birch leaf particles up to concentration 5 mg C L^−1^. We compared somatic growth of *Daphnia* in these treatments to find out does birch leave particles enhance growth under low availability of autochthonous carbon. This experiment was carried out in 40 mL vials. At the end of the experiment the size of *Daphnia* was measured under a microscope, and the individuals were then placed into 1.5 mL Eppendorf^®^ tubes, freeze-dried and stored at −80 °C. Preserved individuals were randomly divided between lipid, fatty acid, carbohydrate and stable isotope analyses. Total biomass growth rate (g) of pooled *Daphnia* for each treatment were calculated as g = (lnBt_21_ − lnBt_0_)/t, where B is biomass (dry weight) at the end (t_21_) and at the beginning (t_0_) of the experiment.

### Lipid, fatty acid and sterol analyses

Lipids were extracted with chloroform:methanol:water (2:1:0.75) from freeze-dried, homogenized cladocerans (0.1–1 mg), terrestrial matter (3–7 mg) and phytoplankton (1–4 mg) samples. The organic phases were pooled, chloroform evaporated off under nitrogen and lipids were measured gravimetrical difference in smooth wall tin capsules. Fatty acid samples were transmethylated with 1% H_2_SO_4_ in methanol and FA methyl esters were run with a gas chromatograph equipped with a mass spectrometer (GC-MS, Shimadzu Ultra) at University of Jyväskylä or at University of Helsinki. Both instruments were equipped with an Agilent^®^ DB-23 column (30 m × 0.25 mm × 0.25 μm), under the following temperature program: 60 °C for 1.5 min, then the temperature was increased at 10 °C min^−1^ to 100 °C, followed by 2 °C min^−1^ to 140 °C, and 1 °C min^−1^ to 180 °C and finally heated at 2 °C min^−1^ to 210 °C and held for 6 min^6^. Sterols were silylated and analyzed with a gas chromatograph (Shimadzu) equipped with a mass detector using Phenomenex ^®^ (Torrance, California, USA) ZB-5 Guardian column (30 m × 0.25 mm × 0.25 μm) and previously published temperature program^71^.

### Klason lignin analysis

Klason lignin content of terrestrial particulate matter and phytoplankton was determined by two-step strong acid hydrolysis with sulfuric acid according to National Renewable Energy Laboratory^72^. In the first stage, about 0.01–0.3 g of sample and 3 ml of 72% H_2_SO_4_ was added to tube and the tubes were placed in a water bath at temperature 30 °C. The samples were stirred during the treatment. After the hydrolysis, the acid was diluted to a 4% concentration by adding 84 ml Millipore-grade water. In the second stage the sample tubes were autoclaved for 1 h in pressure 1 bar (at 121 °C). After autoclaving the samples were separated using sinter glasses (ROBU, 42 mm, 30 mL) and vacuum filtrate system. The sinter glasses with the precipitate were dried at 105 °C for 12 h and weighted, which after sinter glasses were burnt at 550 °C for 3 h, cooled and weighted again. Klason-lignin content was calculated as difference of sinter glasses after filtering and dried at 105 °C and burnt at 550 °C.

### Carbohydrate analyses

Total carbohydrate content was measured using Dubois *et al.*^73^ protocol in which glucose is dehydrated to hydroxymethyl furfural in hot acidic medium. Practically, 0.1–1 mg of freeze-dried *Daphnia*, terrestrial organic matter or phytoplankton was diluted with 1 mL of deionized (MQ) water and 1 mL of 5% phenol solution and 5 mL of sulphuric acid (96% reagent grade) was added into the vials. These were shaken well and placed in 20 °C water bath for 20 minutes. Carbohydrates were measured with a Shimadzu UV-240 spectrophotometer at 490 nm and quantified against calibration curve with glucose (Sigma-Aldrich, 0.05; 0.1; 0.2; 0.5; 0.7; 1 μg μL^−1^). Additionally, we measured contribution (area %) of different types of monosaccharaides based on Laboratory Analytical Procedure (LAP) for determination of carbohydrates in algal biomass (N-REL 2013). For the determination of monosaccharides we used HPLC (Shimadzu) and RID detector (RID-20, Shimadzu) using Phenomenex Relex RHM-Monosaccharide H^+^ (8%) column (size 300 mm × 7.8 mm). Deionized water was used as an eluent with a flow rate of 0.6 mL min^−1^, the running temperature was constantly at +85 °C and the running time was 30 min per sample. The instrument was calibrated using five standards mixed of l-rhamnose, ribose, fructose, mannose, xylose, glucose, galactose and arabinose (Sigma-Aldrich) diluted to deionized water (containing each of 0.0313, 0.125, 0.25, 0.5, 0.75 and 1 g/l). The column could not separate xylose, rhamnose, galactose and mannose from each other, but they eluted at the same time.

### Protein content

Protein content was calculated by conversion elemental nitrogen to protein from equation (N-REL):


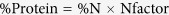


where %N is elemental nitrogen content determined by combustion (Carlo-Erba Flash 1112 series Element Analyzer) and Nfactor is the specific conversion factor (nitrogen content of proteins) for phytoplankton, terrestrial matter and zooplankton. Here, we used 4.78 for phytoplankton and t-POM^74^ and 6.3 for zooplankton^75^.

### Stable isotope analyses

Approximately 0.2–0.6 mg of zooplankton and ≈1.0 mg of phytoplankton and t-POM were weighed in tin cups for δ^13^C and δ^15^N analyses, which were carried out on a Carlo-Erba Flash 1112 series Element Analyzer connected to a Thermo Finnigan Delta Plus Advantage IRMS at the University of Jyväskylä, Finland. These samples were compared to the NBS-22 standard using fish muscle as a laboratory-working standard. The precision of the δ^13^C and the δ^15^N analyses were 0.2‰ and 0.3‰, respectively, for all samples.

### Carbon content of biomolecules

The final results of lipids, proteins, carbohydrates and fatty acids were converted to percent (%) of total organic carbon (TOC) to be able to calculate the contribution of different biochemical groups based on carbon isotope results. This means that stable isotope carbon signal contains only carbon of lipids, proteins, carbohydrates and fatty acids. Therefore, we converted concentrations of the biomolecules to carbon content. The most common biochemical compounds were used to calculate the carbon content of each biochemical group. Lignin carbon content of trees is usually 60–65%, and here we used the average value of 63.9% 76. For carbohydrates, we used the carbon content of glucose which is 40%. Carbon content of fatty acids with 14–22 carbon chain length varies between 75–80%, the proportion being the highest in highly polyunsaturated fatty acids (e.g. DHA). Carbon content of lipids (e.g. triaglycerol, phosphatidylcholine, phosphatidylethanolamine) varies between 69–77%, and we used the average value of 63% for the above mentioned lipids when fatty acid chain length was estimated to be 16.We used 46% as carbon percentage for both proteins and amino acids in this study^75^.

### Fatty acid calculations of batch experiment

We calculated the proportions (mean ± SD) of different FA sources in *Daphnia* in the mixed diet treatments originating from terrestrial particulate organic carbon (t-POC), bacteria and phytoplankton by comparing the actual *Daphnia* FA profiles to hypothetical *Daphnia* FA profiles^12^. A hypothetical FA profile for a mixed diet was calculated = X × (the percentage of total FAs for a particular FA in the 100% *Cryptomonas* diet) + (1 − X) × (the percentage of FAs for a particular FA in the 100% bacterial or t-POC diet). We then compared this hypothetical FA profile to the *Daphnia* FA profile for the t-POC or algal diet and used the Solver function in Microsoft Excel to find the value of X that minimized the Error Sum of Squares between these two profiles. We also used Excel Solver to find the value of X that maximized the fit (*r*^*2*^) between the predicted and observed FA profiles.

### Isotope modeling for experiments

The contribution of ingested phytoplankton and t-POM in *Daphnia* was calculated using δ^13^C values of the diet in both life table and batch experiments. Mean (±SD) carbon assimilation based on δ^13^C values, was calculated with IsoError software (version 1.04; ref. 77). In all cases we had only two diet sources and, thus, the uncertainty caused by variability of both sources was taken into account.

### Statistical analyses

The differences in the biochemical composition of diet sources or Daphnia fed with different diets (Cryptophytes, Green algae, Reed, Birch) were tested using 1-way ANOVA, or if normality assumptions were not met, using Kruskal-Wallis test. Pairwise comparisons were conducted with least significant difference test. The relationships between proportions of carbohydrates, fatty acids or proteins in Daphnia and the degree of allocthtony were examined using linear (x + b) or nonlinear (y = ae^−xb^) regression models. Similarly, the relationships between the proportion of terrestrial carbon (t-POC) in the diet and the proportion of assimilated terrestrial-origin carbon by *Daphnia* (estimated with stable carbon isotope or fatty acid analyses) were examined using linear or nonlinear (y = ax^b^) regression models.

Growth response and dietary thresholds of *Daphnia* in the experiments were estimated with nonlinear regression analysis. The model was modified from von Bertalanffy growth equation^78^, describing somatic growth rates (*Wg* mg DW d^−1^) in relation to the proportion of autochthonous carbon in the diet:


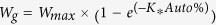


where *W*_*max*_ represents the maximum growth rate, *K* = von Bertalanffy growth coefficient and *Auto*% the proportion of autochthonous carbon in the diet. Because the *W*_*max*_ cannot be used for estimating growth saturation, we used 90% growth rate estimates^79^.

### Cladoceran sample collections

Herbivorous cladoceran (mostly *Daphnia*) samples were collected from lakes in southern and eastern Finland and subarctic Finnish Lapland between May and September during several years (Table 2). The lakes were classified as brown-water, clear-water, subarctic and eutrophic lakes based on dissolved organic carbon (DOC), total phosphorus (TP) concentration and geographical location; brown-water lakes: DOC > 10 mg C L^−1^, TP < 35 μg P L^−1^, 61–62°N; clear-water lakes DOC < 10 mg DOC L^−1^, TP < 35 μg P L^−1^, 62°N; subarctic lakes DOC < 10 mg DOC L^−1^, TP < 20 μg P L^−1^, 68–69°N and eutrophic lakes DOC < 10 mg DOC L^−1^, TP > 35 μg P L^−1^, 60–61°N. Zooplankton samples were collected with vertical or horizontal tows of zooplankton net with mesh size of 25 μm. Total phosphorus (TP), total nitrogen (TN), chlorophyll *a* and dissolved organic carbon (DOC) concentration was analyzed using validated routine methods of the Finnish Standard Association ( htpp://www.sfs.fi/en/). The pigments were extracted in ethanol^80^ and measured with a Shimadzu UV-240 spectrophotometer at 665 nm and 750 nm for chlorophyll *a*.

### Fatty acid based modeling of field collected cladocerans

To generate estimates of dietary resource assimilation by zooplankton of different basal resources, we used the Bayesian mixing model FASTAR^43^,^81^, which is adapted for analysis of fatty acids from the isotope mixing models MixSIR^82^ and SIAR^83^. The model uses a ‘resource library’ file consisting of means ± sd fatty acids of *Daphnia* fed a diversity of known basal monocultures in controlled laboratory feeding trials^43^,^79^. Each distinct end member (points in Fig. 5A) is a unique mean fatty acid profile of *Daphnia* in a fully replicated feeding trial fed one algal taxon from the nine potential basal resource groups considered here. The available phytoplankton, DOC content, and bacteria observed in these lakes was initially used to determine which end members would need to be experimentally fed to *Daphnia* in the feeding trials to establish the resource library (e.g. 43, ref. 84). The mixing model aggregates the unique species fatty acid profiles to a single ‘group-level’ source (e.g., cryptophytes) as the mean of the fatty acid profiles for *Daphnia* fed the different cryptophyte taxa. Of the bacterial groups we included Actinobacteria, which generally represent ~30% of heterotrophic bacteria in boreal lakes^85^,^86^, and methane-oxidizing bacteria (MOB), important in small stratified lakes, in the model. The third abundant bacterial group in boreal lakes, *Polynucleobacter*, was not included in the model because in previous laboratory experiments these bacteria proved to be toxic to *Daphnia*^37^.

Uncertainty in the model sources at the group level is accounted for by using the calculated standard deviations of fatty acid values across diets within a given phytoplankton group^81^. Our analysis makes the general assumption that, at this group scale, we are accounting for all of the important potential prey items for *Daphnia* in the lakes studied. This is a reasonable assumption because the available phytoplankton, DOC and bacteria have been identified for these lakes, and most importantly, potentially missing individual basal resource groups are expected to group largely according to taxonomy^6^,^87^,^88^. This means that even if a particular individual taxon was not included in the model, the group is adequately characterized with multiple end members that are representative of the group mean ± sd fatty acid values. Moreover, it is evident that the sampled wild zooplankton from all lakes fall inside of the multivariate resource-polygons of the resource library of the fatty acid profiles of *Daphnia* fed end members in controlled feeding trial (Fig. 5A).

As the resource library natively accounts for trophic modification of fatty acids by the consumer, the model does not assume universal or non-species specific trophic fractionation constants^43^,^58^. This means that, in practice, the resource library is the fatty acid profiles of *Daphnia* fed the known diets (Fig. 5A) rather than just the values the raw phytoplankton FA profiles^43^,^81^. For this analysis, FASTAR differed from Galloway *et al.*^43^ in that SIAR was used as the underlying model, and the model was run with an expanded nine-source resource library, using all 24 fatty acids to solve likely dietary contribution of these resources to Cladocerans in all 21 study lakes. The posterior distributions (Fig. 6) were estimated using the Gibbs sampling algorithm of Markov Chain Monte Carlo (MCMC) in R ref. 89 implemented as described in Galloway *et al.*^43^. The model was run for each individual lake, and in addition to showing the full posterior distributions (Fig. 6) we report the median model solution for each lake, with summaries by lake type computed from the individual lake medians. Resource group median FASTAR model results were summed for the post-hoc summary comparisons of high, medium, and low quality resources by lake type.

The comparisons of zooplankton resource assimilation of the different food quality resources (high: cryptophytes, diatoms, dinoflagellates; medium: green algae and golden algae; and low: t-POM, Actinobacteria, MOB) were made using one-way ANOVA followed by Hochberg’s GT2 posthoc tests (due to unequal sample sizes between treatments) using SPSS v. 19. Principal components analysis^89^ was used to visualize the multivariate fatty acid resource-library of experimentally fed *Daphnia* fed known basal end-members and the wild cladocerans collected in the study lakes.

## Additional Information

**How to cite this article**: Taipale, S. J. *et al.* Terrestrial carbohydrates support freshwater zooplankton during phytoplankton deficiency. *Sci. Rep.* **6**, 30897; doi: 10.1038/srep30897 (2016).

## Figures and Tables

**Table 1:** Klason lignin, carbohydrate, protein and lipid contribution of total organic carbon (TOC) in phytoplankton and in leaves of terrestrial trees and reed (na = not available).

Common name	Functional Group	Strain	Media	Klason lignin	Carbohydrates	Proteins	Lipids
	Phytoplankton			Mean ± SD	Mean ± SD	Mean ± SD	Mean ± SD
Green algae	*Acutodesmus* sp.	University of Basel	MWC^64^	30.2 ± 0.3	18.8** ± **2.6	40.0** ± **0.0	11.0** ± **1.4
	*Monoraphidium griffithii*	NIVA-CHL 8	MWC^64^	na	11.6** ± **1.9	31.9** ± **0.6	13.3** ± **7.4
	**Average**			**30.2 ± 0.3**	**15.2 ± 5.1**	**36.0 ± 5.7**	**12.2 ± 1.6**
Cryptophytes	*Cryptomonas ozolinii*	UTEX LB 2782	AF6 64	26.0	4.4** ± **1.1	50.6** ± **3.0	14.1** ± **2.5
	*Cryptomonas erosa*	CPCC 466	AF6 65	na	8.2** ± **0.5	48.3** ± **1.9	5.9** ± **0.3
	*Rhodomonas minuta*	CPCC 344	Z8 66	na	7.8** ± **1.3	na	na
	*Rhodomonas lacustris*	NIVA 8/82	Z8 66	na	8.7** ± **0.1	44.2** ± **1.2	6.7** ± **2.4
	**Average ± SD**				**7.3 ± 2.0**	**47.7 ± 3.2**	**8.9 ± 4.5**
Diatoms	*Diatoma tenuis*	CPCC 62	Chu 10 67	na	13.7** ± **2.2	38.1** ± **0.4	18.2** ± **2.0
	*Navicula pellicosa*	UTEX B 664	Chu 10 67	na	10.9** ± **0.9	43.8** ± **5.4	9.2** ± **3.3
	**Average ± SD**				**12.3 ± 2.0**	**41.0 ± 4.0**	**13.7 ± 6.4**
Dinoflagellates	*Peridinium cintum*	SCCAP K-1721	MWC^64^	na	9.6** ± **0.7	33.8** ± **0.6	5.5** ± **2.4
Cyanobacteria	*Limnothrix planktonica*	NIVA-CYA 107	MWC^64^	na	13.5** ± **9.1	46.0** ± **1.1	3.6** ± **0.0
	*Pseudanabaena limnetica*	NIVA 276/11	MWC^64^	na	7.1** ± **1.4	57.5** ± **0.9	4.7** ± **0.5
	*Synechococcus elongatus*		MWC^64^	na	na	na	3.4** ± **0.7
	**Average ± SD**				**10.3 ± 4.5**	**51.8 ± 8.1**	**4.2 ± 0.7**
Chrysophytes	*Mallomonas caudata*	*Lake Horkkajärvi*		na	na	39.6** ± **0.7	18.2** ± **2.7
**Leaves of terrestrial trees**							
Arctic Birch	*Betula pubescens subsp. czerepanovii*	*Kilpisjärvi, Finland*		60.0 ± 2.4	23.6** ± **2.6	12.4** ± **0.6	3.3** ± **0.8
Silver Birch	*Betula pendula*	*Jyväskylä, Finland*		70.3 ± 0.5	27.6** ± **0.9	12.6** ± **0.84	12.6** ± **0.8
Red alder	*Alnus rubra*	*Seattle, USA*		39.8 ± 2.0	26.1	12.4** ± **0.6	2.6** ± **0.1
Black cottonwood	*Populus trichocarpa*	*Seattle, USA*		32.8 ± 2.0	33.5** ± **4.9	na	na
Willow	*Salix* spp.	*Seattle, USA*		46.0±	27.5	na	na
Bigleaf maple	*Acer macrophyllum*	*Seattle, USA*		63.8 ± 0.7	37.7** ± **12.9	na	na
	**Average ± SD**			**52.1 ± 14.8**	**29.3 ± 5.2**	**12.5 ± 0.1**	**6.2 ± 5.6**
Reed	*Phragmites australis*	*Joensuu, Finland*		39.2 ±0.1	39.0** ± **1.1	16.9** ± **0.0	1.5 ± 0.1

**Table 2:** Brown-water, clear-water, subarctic and eutrophic lakes were sampled between 2006 and 2015.

Lake	Latitude (°N)	Longitude (°E)	Month and year	Zooplankton	Fraction	DOC (C mg/l)	tot N μg/l	tot P μg/l	Chl α μg/l	Phytop (%)	MOB (%)	t-POM (%)	Actino (%)
Nimetön	61.220	25.190	May 2006	*Daphnia longispina*	PLFA	22.3	828	31	8.5	66.7	0.3	17.4	1.2
Nimetön	61.220	25.190	May 2006	*Daphnia* + *Holopedium*	PLFA	22.3	828	31	8.5	55.9	0.3	15.4	1.4
Mekkojärvi	61.230	25.140	May 2006	*Daphnia longispina*	PLFA	26.3	419	17	11.3	75.0	1.5	6.3	1.0
Mekkojärvi	61.230	25.140	Jul 2006	*Daphnia longispina*	TFA	22	419	17		60.7	15.1	11.5	3.1
Mekkojärvi	61.230	25.140	Oct 2006	*Daphnia longispina*	PLFA	17.6	786	27	12.9	55.6	5.3	14.0	4.0
Horkkajärvi	61.210	25.150	Aug 2013	*Daphnia*	TFA	31.6	766	17	13.8	47.9	13.5	4.8	17.4
Harkkojärvi	62.960	31.040	Aug 2013	*Daphnia*	TFA	16.6	554	18.3	8.2	67.1	0.5	9.1	10.4
Harkkojärvi	62.960	31.040	Aug 2013	*Daphnia*	TFA	16.6	554	18.3	8.2	52.0	0.9	27.4	12.8
Harkkojärvi	62.960	31.040	Aug 2013	*Daphnia*	TFA	16.6	554	18.3	8.2	57.3	0.2	19.4	9.8
Nuorajärvi	62.680	31.120	Aug 2013	*Daphnia*	TFA	16.1	478	22	6.1	55.7	0.4	20.8	20.4
Vähä-Valkjärvi	61.190	25.090	Aug 2011	*Bosmina*	TFA	6.0	581	17	19.9	72.4	0.4	12.0	3.9
Valkea Mustajärvi	61.220	25.120	Aug 2011	*Daphnia*	TFA	5.1	349	12	16.3	50.2	0.3	17.1	2.8
Valkea Mustajärvi	61.220	25.120	May 2006	*Holopedium, Bosmina*	PLFA	5.1	367	8	3	85.8	0.2	10.1	1.3
Syrjänalunen	61.190	25.140	Aug 2013	*Daphnia*	TFA	2.5	271	7	3.1	82.7	1.8	3.0	7.7
Iso Valkjärvi	61.190	25.110	Aug 2013	*Daphnia*	TFA	4.5	402	12	6.4	64.5	0.4	11.4	6.4
Ylinen	62.600	30.220	Aug 2013	*Daphnia*	TFA	6.9	458	7	2.3	72.6	0.4	15.4	5.1
Ylinen	62.600	30.220	Aug 2013	*Daphnia*	TFA	6.9	378	3.6	2.3	48.4	0.4	9.4	29.5
Kuorinka	62.620	29.420	Aug 2013	*Daphnia*	TFA	2.8	190	1.6	1.3	46.9	0.3	9.6	11.1
Kuorinka	62.620	29.420	Aug 2013	*Daphnia*	TFA	2.8	190	1.6	1.3	85.6	0.2	4.2	4.7
Kermajärvi	62.430	28.720	Aug 2013	*Daphnia*	TFA	8.0	417	4.6	2.3	77.1	1.5	5.0	1.9
Oikojärvi	68.500	21.130	Sept 2011	*Daphnia*	TFA	5.6	215	7	2.6	60.3	0.3	12.7	5.5
Oikojärvi	68.500	21.130	Sept 2011	*Daphnia*	TFA	5.6	215	7	2.6	51.4	0.3	14.7	16.1
Ropijärvi	68.410	21.350	Sept 2011	*Daphnia*	TFA	5.4	273	10	2.9	76.0	0.2	16.2	4.5
Kuohkimajärvi	69.030	20.330	Sept 2012	*Daphnia*	TFA	3.0	269	18	1.3	41.8	0.3	13.9	40.0
Kuohkimajärvi	69.030	20.330	Sept 2012	*Daphnia*	TFA	3.0	269	18	1.3	65.3	0.2	8.6	23.2
Siilasjärvi	69.040	20.450	Sept 2012	*Daphnia*	TFA	1.9	159	5	1.2	73.0	0.4	11.7	2.0
Kilpisjärvi	69.000	20.490	Sept 2012	*Daphnia*	TFA	2.8	351	9	0.9	74.4	0.3	17.1	3.0
Lovonjärvi	61.080	25.030	Jul 2015	*Daphnia, Bosmina*	TFA	13.5	785	49	24.4	89.1	0.7	3.4	2.6
Kyynäröinen	60.870	24.170	Jul 2015	*Cladocerans*	TFA	16.9	965	77	44.9	89.9	1.3	2.6	4.4
Kataloistenjärvi	61.020	24.940	Jul 2015	*Cladocerans*	TFA	8.9	620	35	11.1	80.5	0.4	2.3	9.7
Tuusulanjärvi	60.400	25.100	Jul 2015	*Daphnia*	TFA	9.2	940	69	29.0	82.7	0.7	5.5	4.9

Some of them were sampled several times or at 2–3 sites. Mean values of dissolved organic carbon (DOC), total nitrogen (tot N), total phosphorus (tot P) and chlorophyll *a* content (Chl *a*) are also shown. Cladoceran zooplankton consisted of different species (*Daphnia*, *Holopedium* and *Bosmina*). Total fatty acids (TFA) or phospholipids fatty acids (PLFA) were analyzed from zooplankton. FASTAR results of median contribution (%) of phytoplankton (Phytop), methane oxidizing bacteria (MOB), terrestrial particulate organic matter (t-POM) and Actinobacteria (Actino) are presented here.
